# The Cellular Chaperone Heat Shock Protein 90 Is Required for Foot-and-Mouth Disease Virus Capsid Precursor Processing and Assembly of Capsid Pentamers

**DOI:** 10.1128/JVI.01415-17

**Published:** 2018-02-12

**Authors:** Joseph Newman, Amin S. Asfor, Stephen Berryman, Terry Jackson, Stephen Curry, Tobias J. Tuthill

**Affiliations:** a The Pirbright Institute, Pirbright, Surrey, United Kingdom; b Department of Life Sciences, Imperial College London, London, United Kingdom; Loyola University Medical Center

**Keywords:** foot-and-mouth disease virus, hsp90, picornavirus, polyprotein processing, virus assembly

## Abstract

Productive picornavirus infection requires the hijacking of host cell pathways to aid with the different stages of virus entry, synthesis of the viral polyprotein, and viral genome replication. Many picornaviruses, including foot-and-mouth disease virus (FMDV), assemble capsids via the multimerization of several copies of a single capsid precursor protein into a pentameric subunit which further encapsidates the RNA. Pentamer formation is preceded by co- and posttranslational modification of the capsid precursor (P1-2A) by viral and cellular enzymes and the subsequent rearrangement of P1-2A into a structure amenable to pentamer formation. We have developed a cell-free system to study FMDV pentamer assembly using recombinantly expressed FMDV capsid precursor and 3C protease. Using this assay, we have shown that two structurally different inhibitors of the cellular chaperone heat shock protein 90 (hsp90) impeded FMDV capsid precursor processing and subsequent pentamer formation. Treatment of FMDV permissive cells with the hsp90 inhibitor prior to infection reduced the endpoint titer by more than 10-fold while not affecting the activity of a subgenomic replicon, indicating that translation and replication of viral RNA were unaffected by the drug.

**IMPORTANCE** FMDV of the Picornaviridae family is a pathogen of huge economic importance to the livestock industry due to its effect on the restriction of livestock movement and necessary control measures required following an outbreak. The study of FMDV capsid assembly, and picornavirus capsid assembly more generally, has tended to be focused upon the formation of capsids from pentameric intermediates or the immediate cotranslational modification of the capsid precursor protein. Here, we describe a system to analyze the early stages of FMDV pentameric capsid intermediate assembly and demonstrate a novel requirement for the cellular chaperone hsp90 in the formation of these pentameric intermediates. We show the added complexity involved for this process to occur, which could be the basis for a novel antiviral control mechanism for FMDV.

## INTRODUCTION

The Picornaviridae are a diverse family of viruses with icosahedral capsids and a positive-sense, single-stranded RNA genome. They are generally considered to be nonenveloped, although some may acquire an envelope as an alternative means of transmission (1, 2). The picornavirus family contains important pathogens of humans and animals, including poliovirus and human rhinovirus in the Enterovirus genus and foot-and-mouth disease virus (FMDV) in the Aphthovirus genus. FMDV infects multiple livestock and wildlife species and is a significant global economic burden and threat to food security. Foot-and-mouth disease is characterized by vesicle formation around the mouth and on the feet, fever, lameness, abortion, and occasionally death of young animals in severe cases (3).

**FIG 1:**
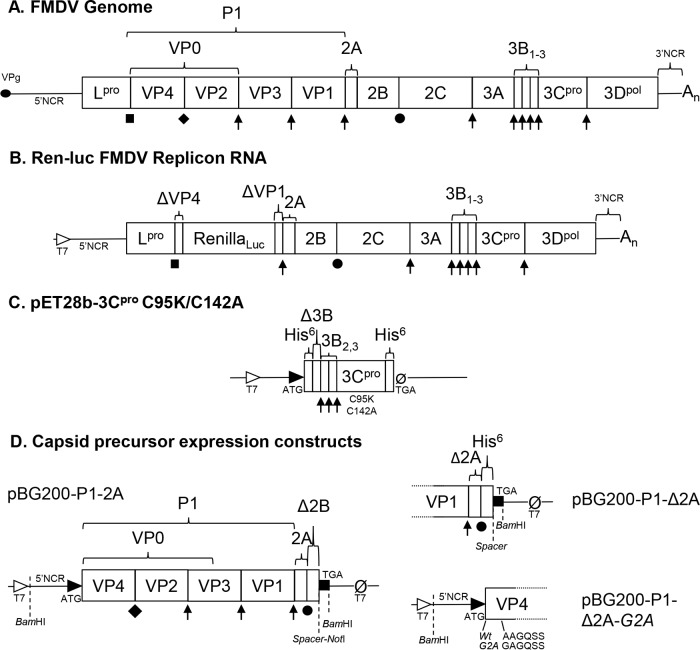
FMDV genome and expression constructs. (A) Representation of the FMDV genome, showing mature protein products in boxes, noncoding regions (NCR), and viral protein genome-linked (VPg), represented with an oval. (B) Subgenomic replicon based upon the O1K FMDV sequence encoding renilla luciferase in place of the majority of the capsid. (C) pET28b plasmid expression constructs encoding 3C protease with C95K/C142A solubility mutations (50). (D) Plasmid expression constructs encoding full-length capsid precursor (P1-2A), capsid precursor with only the cleavage recognition sequence at the start of 2A (P1-Δ2A), and P1-Δ2A encoding a G2A mutation in VP4 to prevent myristoylation (P1-Δ2A G2A). All capsid precursor constructs were under the control of a T7 promoter in the pBG200 backbone (11). Protein processing key: ↑, cleavages performed by 3C^protease^; ●, ribosomal skip; ■, autocatalytic cleavage by L^protease^; ◆, maturation cleavage.

The picornavirus genome encodes both structural and nonstructural proteins which are translated from a single open reading frame as a polyprotein. In FMDV, cotranslational processing results in the generation of a capsid precursor, P1-2A (FMDV genome structure [Fig. 1A]). This capsid precursor is proteolytically processed by a virally encoded protease, 3C viral protease (3C^pro^), to form cleavage products VP0, VP1, and VP3, which remain associated as the protomer, the basic subunit of capsid assembly (4). Five protomers multimerize to form the pentameric capsid intermediate, 12 of which further assemble into the viral capsid (5, 6). If RNA is encapsidated during the assembly process, VP0 is cleaved into VP2 and VP4 and infectious virions are formed; however, capsids lacking RNA (empty capsids) have also been shown to form in cells infected by many picornaviruses, including FMDV (6–10). Recombinant empty capsids can be generated through the expression of the capsid precursor along with 3C protease in heterologous systems (11–14).

The formation of empty capsids from pentamers is often referred to as a self-assembly process, as it has been demonstrated that a sufficient concentration of pentamers is all that is necessary for capsids to form (15, 16), whereas the assembly of pentameric capsid subunits is more complex. In many picornaviruses, including FMDV, the capsid precursor is N-terminally myristoylated by host enzymes (17), and this is thought to provide stability to subsequent assembly steps (11, 18–20) by forming interprotomer interactions (19) essential for the assembly of infectious virions. It is also accepted that precursor processing by 3C^pro^ is an essential step in morphogenesis (6, 18, 21, 22), as the termini of the capsid proteins are separated in the empty capsid (23, 24) and virion (25–28) structures. Processing is thought to enable interactions to occur which stabilize the formation of pentamers (21, 29). While the residues at P1 and P1′ of the 3C^pro^ cleavage sites in the capsid precursor are critical for efficient processing (30), the protease recognition sequences span 8 residues (P4 to P4′), which are thought to be associated with the ability of the enzyme binding cleft to accommodate the capsid precursor substrate (31). However, truncations or mutations to sites distant from the cleavage sites can prevent or impact the efficiency of processing (32–34), suggesting that the capsid precursor is required to be presented in the correct conformation to the viral protease in order for processing to occur efficiently.

Some viruses have been shown to interact with components of the cellular chaperone machinery that facilitates protein folding and homeostasis (35), most likely reflecting a requirement for assisted protein folding as a result of the high rate of production of viral proteins. The growth of many viruses is impaired by inhibitors of hsp90 (36), including several picornaviruses (37–41). Specifically, for several enteroviruses, the ATP-dependent heat shock proteins 70 (hsp70) and hsp90, which are major components of the cellular chaperone machinery, have been shown to interact with P1 (the enterovirus capsid precursor polyprotein) (37, 42). This interaction with hsp90 and cochaperone p23 protects P1 from proteasomal degradation, and it is thought to fold P1 into a conformation allowing it to be processed by the viral protease (37). As described above, processing is a critical event in the assembly of infectious virions. The major capsid protein (VP1) of human and murine noroviruses in the related Caliciviridae family of viruses has also been shown to interact with hsp90, thereby protecting it from degradation (43), suggesting a conserved mode of action for hsp90 between different picornavirus-like virus clades (44).

We previously established cell-free systems to study the assembly of FMDV capsid subunits (18). In this study, we have used established hsp90 inhibitors (45, 46) to show that hsp90 is required for efficient growth of FMDV in cell culture and for P1-2A processing and pentamer assembly in cell-free assays for assembly of capsid subunits.

## RESULTS

### hsp90 inhibition reduces FMDV growth independent of viral RNA replication and protein translation.

To demonstrate a requirement for hsp90 in the life cycle of FMDV, we sought to quantify the effect on viral growth using 17-(dimethylaminoethylamino)-17-demethoxygeldanamycin (17-DMAG), a well-characterized inhibitor of hsp90. 17-DMAG is a water-soluble derivative of geldanamycin (GA), a benzoquinone ansamycin antibiotic which has a structure that is highly similar to the conformation that ATP adopts when occupying its binding site in the hsp90 dimer (47, 48).

**FIG 2:**
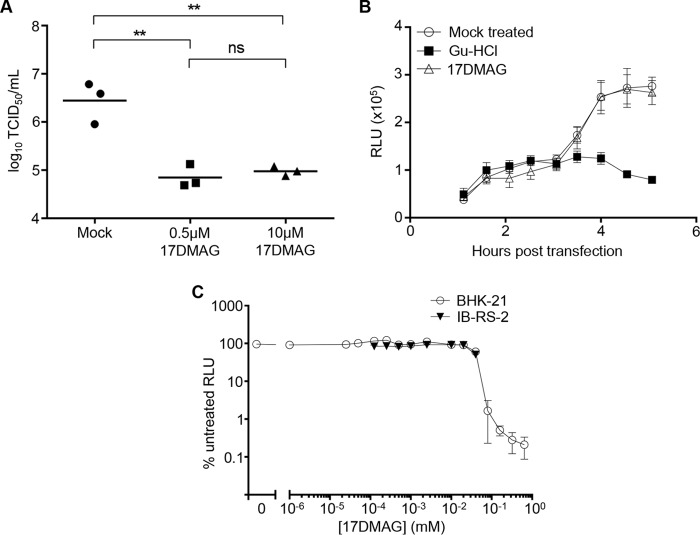
17-DMAG reduces the growth of FMDV in cell culture without affecting translation or replication of the RNA. (A) BHK21 cells were treated with 17-DMAG at the concentrations indicated or mock treated before infection with FMDV strain A22 Iraq. Cells were lysed 8 h postinfection and infectious virus titrated by TCID_50_ assay. Replicates represent independent experiments. (B) IB-RS-2 cells were treated with 17-DMAG (0.5 μM) or GuHCl (3 mM) for 30 min at 37°C before being transfected with FMDV replicon RNA. Luciferase production was measured at half-hour intervals and expressed as relative light units (RLU). Error bars represent the standard deviations from triplicate samples. (C) 17-DMAG cytotoxicity. Cultures of IB-RS-2 and BHK21 cells were treated with a dilution series of 17-DMAG for 8 h at 37°C. Cell viability was determined by quantitating cellular ATP using a luminescence assay (Promega ToxGlo) and luminometer (Hidex Chameleon). Values were converted to percent relative light units (RLU) obtained from mock-treated normal healthy cells. Error bars represent standard deviations from quadruplicate samples.

BHK-21 cells were pretreated with 0.5 μM and 10 μM 17-DMAG prior to infection with FMDV at a multiplicity of infection (MOI) of 1, with drug treatments maintained in the cell culture media throughout the experiment. The cultures were lysed by freeze-thaw after one replication cycle (8 h postinfection), and the levels of infectious virus were quantified by endpoint dilution assay. Treatment with both concentrations of 17-DMAG resulted in a greater than 10-fold reduction in virus titer (*P* ≤ 0.01 by one-way analysis of variance [ANOVA]) compared to the mock-treated control (Fig. 2A).

Previous studies showed that inhibition of hsp90 does not affect the replication of a poliovirus subgenomic replicon RNA (37) or the levels of viral RNA in cells infected by the related enterovirus 71 (40), suggesting that hsp90 does not inhibit viral RNA replication or translation in these systems. To determine if this was also true for FMDV, porcine IB-RS-2 cells were treated with 0.5 μM 17-DMAG or the well-characterized picornavirus replication inhibitor guanidine hydrochloride (GuHCl; 3 mM) (49) or were mock treated before being transfected with an FMDV subgenomic replicon RNA, in which the majority of the capsid coding region was replaced with sequence encoding Renilla reniformis luciferase (as described by Tulloch and colleagues [50] but with luciferase replacing green fluorescent protein [Fig. 1B]). Drug treatments were maintained in the cell culture media throughout the experiment. Luminescence readings were collected between 1 and 5 h posttransfection. No significant differences were observed between 17-DMAG- and mock-treated cells, whereas a significant reduction (*P* ≤ 0.001 by two-way ANOVA) in luminescence signal was observed from 3.5 h onwards in the GuHCl control (Fig. 2B). This result demonstrated that 17-DMAG had no effect on the expression of luciferase from the FMDV replicon, suggesting that RNA replication and translation were unaffected by inhibition of hsp90, and also that the 3C protease activity was not directly affected by the drug.

The cytotoxicity of 17-DMAG was determined for baby hamster kidney (BHK-21) and porcine kidney (IB-RS-2) cell lines to demonstrate that toxicity was not the cause of the reduction in virus titer. A luminescence-based cell viability assay (ToxGlo; Promega) was used and showed the drug to be nontoxic at 20 μM and below (Fig. 2C).

**FIG 3:**
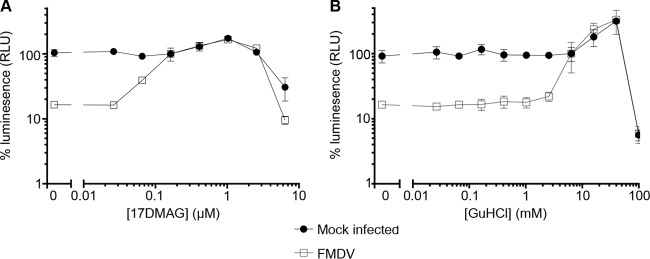
17-DMAG inhibition of virus growth is dose dependent. BHK-21 cells were treated with a 2.5-fold dilution series of 17-DMAG (A) or GuHCl (B). The cells were infected with FMDV at an MOI of 0.01 (□) or mock infected (■) and incubated for 72 h at 37°C. Cell viability was determined by quantitating cellular ATP using a luminescence assay (Promega ToxGlo) and luminometer (Hidex Chameleon). Values were converted to percent RLU obtained from healthy cells. Error bars represent standard deviations from quadruplicate samples.

To demonstrate that FMDV was inhibited by 17-DMAG in a dose-dependent manner, BHK-21 cells were treated with a 2.5-fold dilution series of the inhibitor and subsequently infected with FMDV at low MOI (∼0.01) in a multicycle infection assay. At 3 days postinfection, the ToxGlo viability assay was used to demonstrate the dose at which the cells were protected from viral cytopathic effect (cpe). Infection of the cells in the absence of the inhibitor resulted in extensive cpe (causing an approximately 85% drop in viability in this assay compared to uninfected controls). In contrast, the cells were partially protected at a 17-DMAG concentration of 0.065 μM and fully protected at concentrations of 17-DMAG at 0.163 μM and above (Fig. 3A). To confirm the utility of this assay in determining the antiviral dose response of FMDV, GuHCl was used as a control. Again in the absence of inhibitor, the drop in cell viability due to infection was about 85%, and in this instance GuHCl protected the cells from cpe at concentrations above 2.5 mM (Fig. 3B).

### Capsid precursor processing and pentamer assembly is supported in a cell-free system.

**FIG 4:**
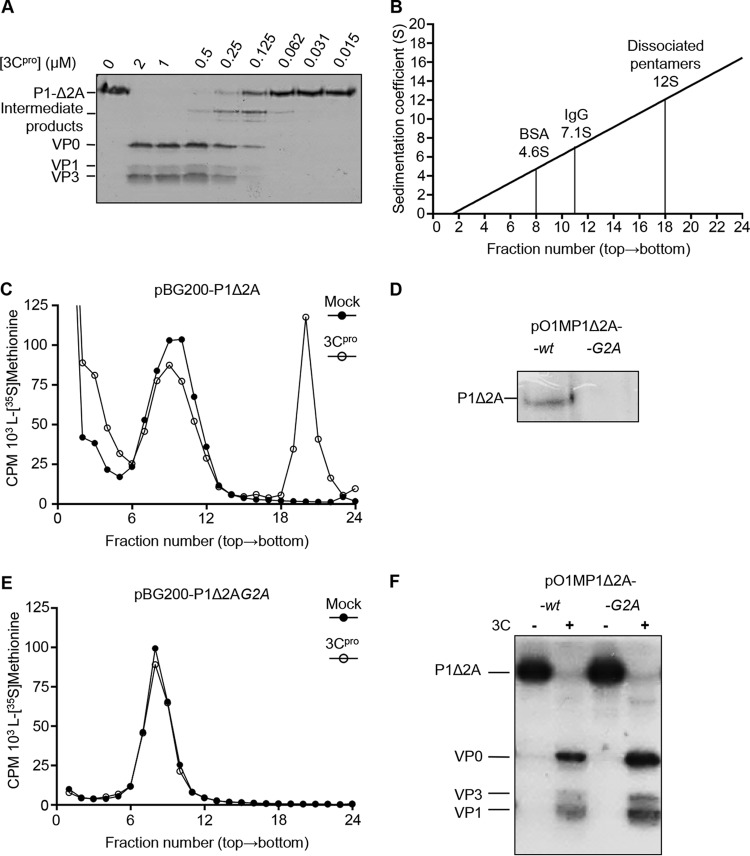
Validation of the cell-free assay for FMDV capsid precursor processing and pentamer assembly. (A) P1-2A processing assay validation. P1-Δ2A capsid precursor generated from expression in rabbit reticulocyte lysates was mixed with a range of concentrations of purified 3C^pro^ for 1 h at 37°C, and the proteins were resolved on 12% SDS-PAGE gels, followed by fluorography. (B) Marker proteins with known sedimentation were separated through 5 to 30% sucrose density gradients, with linear regression analysis used to extrapolate and interpolate unknowns. (C and E) Sucrose density gradient analysis of pentamer assembly reactions with P1-Δ2A-wt (C) and -G2A mutant (E) expression constructs showing mock (●) and 3C^pro^ (○) treatments. (D) Cell-free expression of P1-Δ2A-wt and G2A expression constructs in the presence of [^3^H]myristic acid. (F) Capsid precursor processing assay using P1-Δ2A-wt and -G2A expression constructs labeled with [S^35^]methionine.

Existing evidence points to the involvement of hsp90 in the early stages of assembly of PV and other closely related picornaviruses (37, 40, 42). Therefore, to investigate this part of the FMDV life cycle, a cell-free assembly assay was developed using recombinant proteins. The FMDV 3C^pro^ was generated from a plasmid expression construct (Fig. 1C) in bacteria and purified as previously described (51). A T7 promoter-driven expression plasmid encoding the capsid polyprotein followed by a hexahistidine tag at the C terminus was used to produce radiolabeled capsid precursor protein in rabbit reticulocyte lysates (RRLs). The precursor protein was engineered to contain the first four amino acids of 2A (Δ2A) to retain a native 3C^pro^ cleavage recognition site such that processing would generate the authentic C terminus of P1 (Fig. 1D, pBG200-P1-Δ2A). To ensure complete processing of P1-Δ2A by 3C^pro^, RRLs were programmed with pBG200-P1-Δ2A followed by treatment with a 2-fold dilution series of purified 3C^pro^. This determined that a final 3C^pro^ concentration of 1 μM was sufficient to process P1-Δ2A into the expected products VP0, VP1, and VP3. At lower 3C concentrations, VP0-VP3 and VP3-VP1 intermediates were also observed (Fig. 4A).

The presence of capsid precursors, protomers, and assembled pentamers was determined by sedimentation of samples through sucrose density gradients (SDGs). Proteins with known sedimentation coefficients (BSA 4.6S, IgG 7.1S, and dissociated FMDV pentamers 12S) (52) were used as markers (Fig. 4B). Unprocessed radiolabeled capsid precursor P1Δ2A expressed in RRLs sedimented as a single peak of radioactivity approximately one-third through the gradient (Fig. 4C, solid circles) at the expected position (5S). Samples of capsid precursor that had been processed by 3C^pro^ resulted in a peak at this position, as well as a second peak at approximately two-thirds through the gradient at the expected position of pentamer (14S) (Fig. 4C, open circles). From the processing assays, it was clear that the precursor had been fully processed; therefore, the 5S peak on this gradient likely was protomers (processed capsid precursor) that had not assembled into pentamers. In addition to the analysis of pentamer formation as described above, the assembly reactions were also analyzed in parallel for the assembly of capsids, using different SDG conditions suitable for resolving capsids from smaller precursors. This determined that in the 1-h processing and assembly assay, no empty capsids were formed (data not shown), likely due to pentamers not reaching the threshold concentration required for capsid self-assembly (15, 16). This demonstrated the utility of this assay as a method to specifically analyze the pentamer assembly step. To validate the assay, a mutant construct (Fig. 1D, pBG200-P1-Δ2A G2A) was generated with a VP4 G2A mutation to prevent myristoylation (18, 53, 54). These expression constructs lack FMDV L^pro^, normally found as a self-cleaving protease upstream of VP4, and contain an additional methionine residue to initiate translation at the start of VP4. The G2A notation here takes into account the additional methionine at the N terminus of VP4, which is removed by host enzymes to reveal the myristoylation signal (55). As expected, this mutation successfully prevented myristoylation in RRLs (Fig. 4D) and completely prevented pentamer formation (Fig. 4E), but did not prevent processing, of P1Δ2A (Fig. 4F).

### hsp90 inhibition by 17-DMAG reduces processing and pentamer formation in a dose-dependent manner in a cell-free assay.

**FIG 5:**
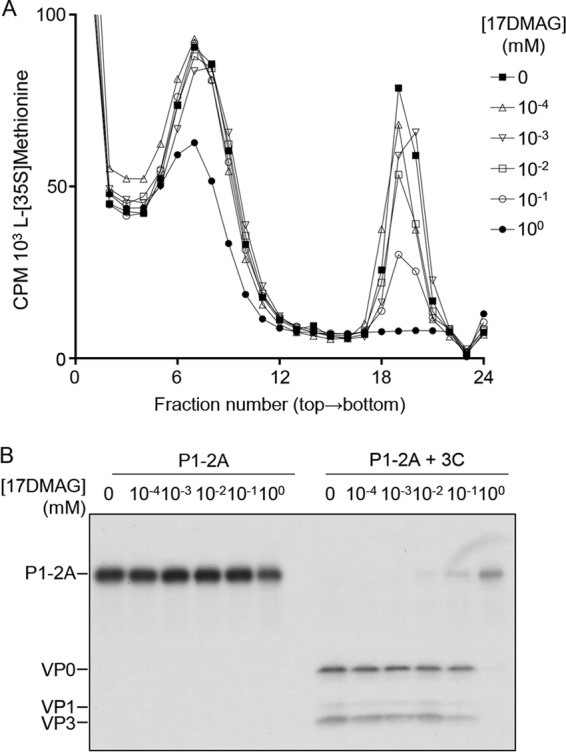
17-DMAG inhibition of hsp90 reduces P1-2A processing and pentamer assembly in a dose-dependent manner. Cell-free expression reactions were pretreated with the concentrations of 17-DMAG indicated prior to expression of radiolabeled P1-2A and processing by addition of purified 3C^pro^. (A) The majority of the samples were sedimented through 5 to 30% (wt/vol) sucrose density gradients and material in gradient fractions detected by scintillation counting. (B) Samples were also removed before sedimentation, resolved through 12% SDS-PAGE gels, and analyzed by fluorography.

The cell-free processing and assembly assay described above was adopted to examine the involvement of hsp90 in pentamer assembly. In these experiments, plasmids expressing capsid precursor with a full-length 2A (Fig. 1D, pBG200-P1-2A) instead of a truncated 2A (P1-Δ2A) were used, in the event that the full-length sequence was required for an appropriate interaction with heat shock proteins to occur. The pBG200-P1-2A plasmid was used to program RRLs that were pretreated with a 10-fold dilution series of 17-DMAG prior to initiation of transcription and translation. In addition to using the drug at nontoxic concentrations, higher concentrations of 17-DMAG (beyond the cell culture toxicity limit) also could be tested in this system. P1-2A then was processed by the addition of recombinant 3C^pro^ and pentamer assembly analyzed by SDG as described above. In the absence of drug, gradient peaks were observed with the expected sedimentation for capsid precursors, protomers (both 5S), and assembled pentamers (14S). In the presence of 17-DMAG, pentamer formation was reduced in a dose-dependent manner, and at a 17-DMAG concentration of 1 mM, pentamer assembly was completely prevented (Fig. 5A). At 10-, 100-, and 1,000-μm drug concentrations, unprocessed P1-2A was also detected in increasing amounts by autoradiography (Fig. 5B), indicating a dose-dependent inhibition of processing under these conditions.

**FIG 6:**
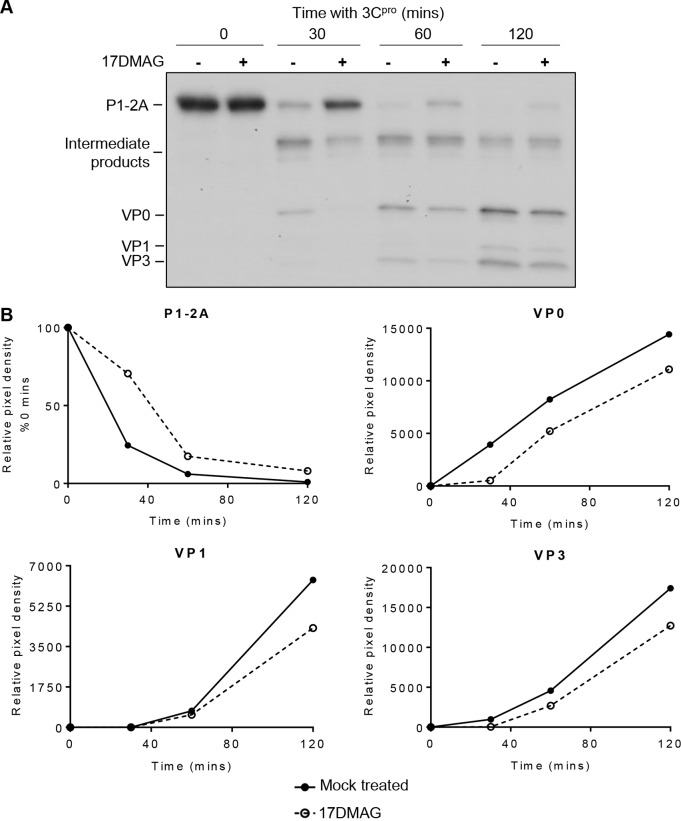
Inhibition of hsp90 reduces the rate of P1-2A processing. (A) Cell-free expression reaction mixtures were pretreated with 10 μM 17-DMAG or mock treated prior to expression of radiolabeled P1-2A and processing by addition of purified 3C^pro^ for the times indicated. Samples were resolved through 12% SDS-PAGE gels and analyzed by fluorography. A reduced concentration of 3C^pro^ was used (0.25 μM compared with 1 μM in a standard assay). Relative band intensity was performed on scanned film images for each cleavage product using NIH ImageJ analysis software (v1.50). (B) Densitometric analysis using ImageJ software was used to generate relative band intensity of the image shown in panel A, and this was plotted for the mock- and drug-treated conditions over time.

To determine whether inhibition of pentamer assembly was a direct result of precursor being unable to multimerize or was caused indirectly by an inhibition of 3C^pro^ processing, reactions (capsid precursor and 3C^pro^) were pretreated with 10 μM 17-DMAG or mock treated, and processing of the polyprotein was assessed at several time points by separating radiolabeled products by SDS-PAGE and detecting by autoradiography. This showed that P1-2A was processed into intermediate precursor products and endpoint products (VP0, VP3, and VP1) as expected and that in the presence of 17-DMAG, this processing appeared to be delayed (Fig. 6A). In these experiments, lower concentrations of 3C^pro^ (0.25 μM) were used to allow the visualization of processing intermediates, ensuring reactions had not reached completion (where potential differences in reaction rates could be masked by analyzing only endpoint products). Autoradiographs were analyzed by densitometry to quantitate the rate of processing. This confirmed that the rate of cleavage of P1-2A into all endpoint products (VP0, VP3, and VP1) was retarded under the drug-treated condition (Fig. 6B), demonstrating that 17-DMAG was affecting the rate of protomer formation.

### An alternative hsp90 inhibitor, luminespib, also inhibits P1-2A processing.

**FIG 7:**
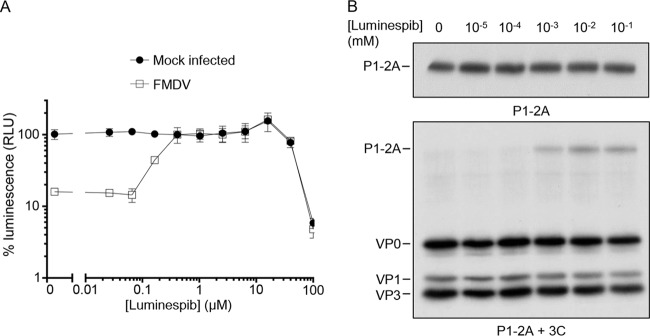
Luminespib inhibits virus growth and reduces P1-2A processing is a dose-dependent manner. (A) BHK-21 cells were treated with a 2.5-fold dilution series of luminespib and then infected with FMDV at an MOI of 0.01 (□) or mock infected (■) and incubated for 72 h at 37°C. Cell viability was determined by quantitating cellular ATP using a luminescence assay (Promega ToxGlo) and luminometer (Hidex Chameleon). (B) Cell-free expression reactions were pretreated with luminespib at the concentrations indicated prior to expression of radiolabeled P1-2A and processing by addition of purified 3C^pro^. The samples were resolved through 12% SDS-PAGE gels and analyzed by fluorography.

In order to confirm that the reduction in FMDV growth was due to the inhibition of hsp90 and not an off-target effect of 17-DMAG, a different class of hsp90 inhibitor was also tested. Luminespib resembles the macrocyclic lactone antibiotic radicicol, which, similar to 17-DMAG (a geldanamycin derivative), binds to the N-terminal domain nucleotide binding pocket of hsp90, inhibiting its ATPase activity and subsequent ability to change conformation to fold client proteins (48). To confirm that this structurally different inhibitor had an effect similar to that of 17-DMAG, cells were treated with a 2.5-fold dilution series of luminespib before infection with FMDV at an MOI of 0.01, and at 72 h postinfection (hpi) cell viability was assessed (as described previously). At concentrations above 0.16 μM the cells were fully protected from cpe-induced loss in viability (Fig. 7A). To confirm luminespib-inhibited P1-2A processing, RRLs expressing P1-2A were pretreated with a 10-fold dilution series of luminespib or mock treated with dimethyl sulfoxide (DMSO), and the processing phenotype was analyzed by autoradiography (Fig. 7B). A dose-dependent inhibition of P1-2A processing was indeed evident at higher concentrations of this drug.

## DISCUSSION

Capsid assembly of enteroviruses has been shown to require hsp90 (37, 38, 40–42), but this is not true of all members of the picornavirus family (56). Here, we have investigated the requirements for FMDV (genus Aphthovirus) assembly and provide evidence of a role for the cellular chaperone hsp90 in the processing of the capsid precursor, which is a vital step required for precursor multimerization into pentameric subunits and capsid assembly. Pharmacological inhibition of hsp90 resulted in dose-dependent reduced viral growth in cell culture without affecting translation or replication of a subgenomic reporter replicon. hsp90 inhibition was shown to reduce P1-2A processing and pentamer formation in a cell-free system, therefore demonstrating that FMDV assembly requires the cellular chaperone machinery.

Picornavirus assembly has previously been demonstrated to proceed through several closely linked stages. For most picornaviruses, the nascent viral polyprotein is cotranslationally myristoylated by host enzymes, and the capsid precursor is separated from the other translation products via proteolysis or a ribosomal “skipping” mechanism. Capsid precursors are then processed by the viral 3C or 3CD protease, facilitating their multimerization into a pentameric capsid subunit and encapsidation of the viral RNA to form virions or empty capsid in the absence of RNA (6). However, it is now clear that this simple description of capsid assembly is incomplete, and recent evidence suggests the requirement for interactions of the picornavirus capsid precursor with chaperones such as hsp90. Inhibition of hsp90 has been shown to lead to the increased turnover of viral proteins from a diverse range of families (36). The capsid proteins of poliovirus and human and murine noroviruses (members of the calicivirus family) (43) have been shown to require hsp90 for the stability of their capsid proteins, and for poliovirus this was attributed to an inhibition of capsid precursor processing (37). Viral capsids are composed of multimeric copies of a single repetitive subunit which must associate correctly to encapsidate the viral genome while retaining the functionality required to enter new cells (57). Due to the multimeric nature of viral capsids, a lack of control during assembly is likely to result in protein misfolding, which can be detrimental to the virus by generating proteins with a dominant-negative phenotype capable of “poisoning” capsid assembly (58). To prevent this, it appears that viruses across different families have developed a requirement for cellular chaperones to maintain the stability of their capsid proteins during assembly. This suggests a common requirement for correctly folded proteins in order for correct capsid processing and assembly to proceed.

In our study, a cell-free assay was used (in rabbit reticulocyte lysates) to specifically analyze the precursor processing and pentamer assembly stages of FMDV capsid assembly. N-terminal myristoylation of the precursor previously has been shown to be required for picornavirus capsid assembly by stabilizing pentamer formation (18, 19), which is vital for efficient capsid formation (53, 59–61). Consistent with these observations, in our system the assembly of capsid precursors into pentamers was prevented by mutation of the myristoylation signal in P1.

The cell-free assay demonstrated a dose-dependent inhibition of both capsid precursor processing and pentamer assembly in the presence of inhibitors of hsp90, which is a critical step in the formation of new FMDV virions. This conclusion is supported by findings that treatment with 17-DMAG and luminespib resulted in a dose-dependent inhibition of virus growth in cell culture. There was a reduction in virus yield of more than one log when cultures were treated with 10 μM 17-DMAG, which contrasted with a 50% reduction in pentamer formation caused by 17-DMAG in the cell-free assay. The reason for the difference in the magnitude of the effect could be due to differences in the nature of the assays. For example, in cells, delays in capsid protein folding result in its degradation (37), which might contribute to the reduction in virus yield.

When the rate of processing was considered in the cell-free assay, it was clear that processing of P1-2A was delayed by the addition of 17-DMAG, suggesting that either hsp90 was not completely inhibited by the drug or that P1-2A was able to fold into a conformation required for processing without hsp90, but that this occurred more slowly or less efficiently in the presence of the drug. Interestingly, in Fig. 5 and 7, when hsp90 was inhibited, processing intermediates were not detected. We interpret this to mean that when functional hsp90 is in short supply, P1-2A may exist as two pools, one where precursor is folded correctly and cleaved rapidly by the high concentration of 3C to leave no processing intermediates and a second pool of precursor molecules not yet folded correctly and which cannot be processed, hence remaining as intact P1-2A.

The canonical model for protein folding in the cytosol is that nascent polypeptides are protected from misfolding by chaperone complexes involving another heat shock protein, hsp70. Some proteins then can be cycled into an additional folding pathway that involves hsp90 (62–66). In the current study, perhaps hsp70 chaperone complexes are sufficient to initiate P1-2A folding into the correct conformation, but this process is more efficient if hsp90 is involved. Interestingly, a previous study identified an interaction between hsp70 and the P1 of enteroviruses (PV and coxsackie virus B1) (42); therefore, treating cell-free assays with inhibitors of the hsp70 system in addition to 17-DMAG may have an additive effect in preventing FMDV P1-2A folding and subsequent processing.

For viral polyproteins that are processed by cleavage at multiple sites, such as in FMDV, there is evidence for the cleavages to occur in a preferred order (67). In addition, in PV, mutations which increased the rate of cleavage at the VP0-VP3 junction resulted in a virus with a reduced growth rate (68). The preferential ordering of multiple cleavages may be controlled by the different sites being cleaved with different efficiencies. Sequential processing may provide a functional benefit to the virus, such as facilitating a subtle conformational alteration required for the next cleavage to occur. In addition, some processing events may involve distant parts of the polyprotein. For example, a recent study identified a substitution at the FMDV P1/2A junction which prevented this cleavage and resulted in the generation of a compensatory mutation distant in sequence and structure from this site (69). Such long-range requirements for cleavage suggest that such distant sites are involved in the interaction between P1-2A and hsp90.

From the results presented here, it is likely that hsp90 increases the efficiency of FMDV capsid precursor folding and subsequent virion assembly. Using hsp90 inhibitors, studies with poliovirus have indicated it is not possible to select viruses with a resistant phenotype (37). A major problem for the control of FMD outbreaks in normally disease-free countries is the delay in onset of immunity following emergency vaccination. Antiviral compounds have the potential to provide vital protection during this delay (70). Preventing or reducing FMDV assembly as demonstrated here by 17-DMAG and luminespib *in vitro* provides a potential antiviral strategy to help control outbreaks until the onset of immunity following vaccination and provides a novel method by which to understand further the FMDV assembly pathway.

## MATERIALS AND METHODS

### Cells, virus, and inhibitors.

FMDV strain O1 Manisa was propagated in baby hamster kidney (BHK-21) and pig kidney (IB-RS-2) cell lines, which were obtained from the central services unit (CSU) at The Pirbright Institute. BHK-21 cells were grown in Glasgow's minimal essential medium (GMEM; Life Technologies) with 10% fetal bovine serum (FBS), 2 mM l-glutamine, 100 U/ml penicillin, 100 μg/ml streptomycin, and 5% tryptose phosphate broth (TPB; CSU). IB-RS-2 cells were grown in the same medium as BHK-21 cells but with 10% adult bovine serum (ABS; Life technologies) substituting for FBS. During luminescence counting experiments, cells were maintained in Dulbecco's modified Eagle's medium (DMEM) lacking phenol-red indicator (Life Technologies).

hsp90 inhibitors 17-DMAG (CAS no. 467214-21-7; Invivogen) and Luminespib (NVP-AUY922; CAS no. 747412-49-3; Selleckchem) were reconstituted in deionized water or DMSO, respectively, aliquoted, and frozen at −80°C. These stocks of drugs were diluted into cell culture medium or reticulocyte lysate before use.

### Cytotoxicity assay.

Cellular toxicity caused by hsp90 inhibition was measured using a luminescent cell viability assay based on quantitation of ATP (ToxGlo; Promega). Cells were grown in μclear 96-well plates (Greiner Bio-One) and maintained in phenol red-free DMEM (Life Technologies). A dilution series of drug inhibitor was made in phenol red-free medium, and 100 μl replaced the medium in each well. For antiviral dose-response experiments, FMDV was added at an MOI of 0.01. At the end of the toxicity period required, room temperature ATP detection reagent was added to ATP detection substrate and mixed thoroughly, and 100 μl was added to each well. After 10 min, luminescence was detected using a Chameleon V plate reader (Hidex).

### Virus titration.

Virus infectivity was titrated by endpoint dilution. Serially diluted samples were used to infect BHK-21 cells in 96-well plates, and the 50% tissue culture infective dose (TCID_50_) was calculated using the Reed-Muench method (71).

### Subgenomic replicon assay to quantitate replication of FMDV RNA.

Replicon RNA was transcribed *in vitro* from cDNA plasmids in which the majority of the structural proteins had been replaced with a reporter gene, as described by Tulloch and colleagues (49). In this case, the reporter gene encoded renilla luciferase. To perform the assays, IB-RS-2 cells were grown in μclear 96-well plates (Greiner) using phenol red-free DMEM. Medium was removed from the wells and replaced with triplicate conditions of fresh media or drug treatment, and the cells were incubated for 1 h at 37°C. The cells were transfected with 90 ng of FMDV replicon RNA and 1 μl of Lipofectamine 2000 (Thermo) per well according to the manufacturer's instructions. EnduRen live cell substrate (Promega) was prepared in fresh medium (with drug treatments as required) and added to cells on top of the transfection mixtures. Luminescence was read periodically on a Chameleon V plate reader (Hidex).

### Bacterial expression and purification of 3C^pro^.

A pET28b plasmid encoding the Δ3B1-3B2-3B3-3C-His6 sequence from FMDV A_10_61 with two mutations to enhance enzyme solubility (C95K/C142A) was obtained from Stephen Curry at Imperial College London (50). Escherichia coli BL21(DE3) pLysS was transformed with this plasmid and grown to an optical density at 600 nm of 0.4 to 0.7 absorbance units. Expression was induced by the addition of 1 mM isopropyl-β-d-thio-galactosidase (IPTG; Sigma) for 4 h at 37°C. Bacteria were harvested by centrifugation and resuspended in a lysis buffer containing 50 mM HEPES, pH 7.1, 200 mM NaCl, 1× HALT protease inhibitor cocktail (Thermo Fisher Scientific), 1% Igepal CA-630 (Sigma), 100 μg/ml lysozyme, 100 μg/ml DNase (Invitrogen) on ice for 1 h. Lysates were subjected to 12, 30-s sonication cycles at an amplitude of 10 μm (Microson XL-2000; Misonix) with 30 s on ice between each sonication. Lysates were clarified by centrifugation and His-tagged 3C^pro^ purified by nickel ion affinity chromatography using HisTrap columns (GE Healthcare). Elution fractions were analyzed by SDS-PAGE, and fractions with the highest concentration of purified protein were pooled and dialyzed against 50 mM HEPES, pH 7.1, 0.2 M NaCl, 1 mM EDTA, 1 mM β-mercaptoethanol, and 5% glycerol. Purified dialyzed proteins were stored at −80°C.

### Cell-free processing and pentamer assembly assay.

FMDV capsid expression constructs were created using standard molecular biology techniques (72). A plasmid encoding the capsid and 3C protease genome regions of the O1 Manisa strain of FMDV in pBG200 T7 expression vectors (11) were used as a template to generate the plasmids pBG200-P1-2A and pBG200-P1-Δ2A, with the primer sequences described in Table 1, by PCR. The Δ2A versions of P1 were designed to encode the first 12 bases of the 2A protein-coding region followed by a His6 tag to allow purification in other experiments. For the chaperone experiments, it was deemed important to express the complete 2A region, so the pBG200-P1-2A plasmids were generated. PCRs were performed using KOD polymerase (Roche), and a VP0 G2A substitution to prevent myristoylation was introduced into the coding sequence of pBG200-P1-Δ2A to generate pBG200-P1-2A-*G2A* using QuikChange lightning mutagenesis (Agilent) and the primer sequences described in Table 1.

**TABLE 1:** Primer sequences for cloning

Primer name	Nucleotide sequence (5′–3′)
pBG200-P1-2A_*fwd*	TATAGGATCCGTTTAAACTTTCCACAACTGACACG
pBG200-P1-2A_*rev*	ATATGGATCCTCAGCGGCCGCTGGGGG
pBG200-P1-Δ2A_*fwd*	TATAGGATCCGTTTAAACTTTCCACAACTG
pBG200-P1-Δ2A_*rev*	ATATGGATCCTCAGTGGTGGTGGTGGTGGTGTGCAGCAAAATTTAGAA-GCTGTTTCACCGGTG
P1-Δ2A_*G2A_fwd*	GAAAGGTTACCATGGCAGCCGGGCAATCCAG
P1-Δ2A_*G2A_rev*	CTGGATTGCCCGGCTGCCATGGTAACCTTTC

Radiolabeled P1-2A was generated from plasmids using *in vitro* transcription and translation reactions in rabbit reticulocyte lysates (TnT quick; Promega) according to the manufacturer's instructions. Reaction mixtures contained 20 ng/μl plasmid DNA, 8 Bq/μl [^35^S]methionine (EasyTag; PerkinElmer), and various concentrations of hsp90 inhibitor and were incubated at 30°C for 1.5 h. Processing and assembly of P1-2A into pentamers was achieved by the addition of 1 μM 3C^pro^ to the reaction and incubation at 37°C for 1 h. Following incubation, free radiolabel was removed from reactions by dialysis at 4°C against phosphate-buffered saline (PBS), using mini-dialysis units (Slide-A-Lyzer; Pierce) that had been preblocked with 1% bovine serum albumin in PBS.

### SDS-PAGE and fluorography.

Products of processing reactions were separated by SDS-PAGE. Gels were soaked in 1 M sodium salicylate (VWR) for 30 min, dried on a slab gel dryer (DrygelSR) for 1 h at 80°C, exposed to film at −80°C overnight, and developed using standard photographic reagents. Digital images of gels and films were generated using a scanner (Epson). The images that are presented were decolored, and the contrast and brightness settings were adjusted minimally using Adobe Photoshop. Relative band intensity was quantified on scanned film images for each cleavage product using NIH ImageJ software (v.1.5) (73). Signals used to generate data were determined to be within the linear range of the film by comparison of multiple exposures.

### SDGs.

Sucrose density gradients (SDGs) were used to separate capsid precursors and protomers from pentameric capsid components. Gradients were prepared in 5-ml ultracentrifuge tubes (polyclear; Biocomp) using the Gradient Master system (Biocomp). After the gradient was formed, a volume of 240 μl sucrose solution was removed from the top of the gradient, and the samples were diluted to this volume in PBS and layered on top of the sucrose. The tubes were subjected to ultracentrifugation for 6 h at 367,598 × *g* maximum, 286,794 × *g* average, at 10°C in a SW55Ti rotor (Beckman).

Following ultracentrifugation, gradients were fractionated into equal fractions using a Piston Gradient Fractionator (Biocomp). Radioactive SDG fractions were diluted 1:5 in OptiPhase supermix (PerkinElmer) and counted in scintillation vials for 3 min on an LS6500 multipurpose scintillation counter (Beckman).
